# Flame Retardancy and Smoke Suppression of Silicone Rubber Foam with Microencapsulated Sepiolite and Zinc Borate

**DOI:** 10.3390/polym15132927

**Published:** 2023-07-01

**Authors:** Furu Kang, Jiayao Tu, Heng Zhao, Zujin Bai, Tiantian Zhang

**Affiliations:** 1 Postdoctoral Station of Geological Resource and Geological Engineering, Xi’an University of Science and Technology (XUST), Xi’an 710054, China; 2 Shaanxi Key Laboratory of Prevention and Control of Coal Fire, Xi’an University of Science and Technology (XUST), Xi’an 710054, China

**Keywords:** silicone rubber foam, flame retardancy, smoke suppression, microencapsulated sepiolite, zinc borate

## Abstract

The flame-retardant micro-encapsulated sepiolite (MSEP) was successfully prepared by sol-gel method. Fourier transform infrared, X-ray photoelectron spectroscopy, scanning electron microscopy, and energy dispersive spectroscopy were utilized to prove that sepiolite was encapsulated. Then, the mechanical properties, flame retardance, smoke suppression, and pyrolysis characteristics of silicone rubber foam (SiFs) with MSEP and zinc borate (ZB) were analyzed. The results indicated that the tensile and compressive properties of SiFs could evidently improve with the incorporation of MSEP/ZB. SiFs with 3 wt% MSEP and 6 wt% ZB could achieve an limiting oxygen index value of 30.9 vol% and UL-94 V-0 rating, the time to ignition was 64.7% above that of pure SiFs, the peak heat release rate and total heat release were 42.7% and 25.0% lower than that of pure SiFs, respectively. Furthermore, the peak smoke production rate and total smoke production of SiFs with 3 wt% MSEP and 6 wt% ZB were merely 54.22% and 64.10% of pure SiFs. Especially, the thermal stability of SiFs was significantly enhanced, and the carbon residue of SiFs became denser and more complete after adding 3 wt% MSEP and 6 wt% ZB.

## 1. Introduction

Silicone rubber foam (SiFs) is a high-performance and functional polymeric elastomer composed of Si-O-Si bonds as the main chain [1,2,3]. SiFs not only has electrical insulation, non-toxicity, durability in various weather conditions, and chemical stability, but also has the characteristics of lightweight, soundproof, and sound shock-absorbing [3,4], so it is widely used in aerospace, transportation, medicine, national defense, architecture, and other fields [5,6,7]. However, SiFs contains a large number of hydrocarbon groups, it can burn and release vast quantities of smoke when encountering a flame, which limits its promotion and application in high flame-retardant requirement fields [8,9].

To further improve the flame retardancy and smoke suppressing of SiFs, many scholars have carried out relevant research [10,11]. As an effective flame retardant, hydrotalcites were often used to improve the flame retardancy of SiFs. Compared with pure SiFs, the heat release rate (HRR) and total smoke production (TSP) of SiFs with 3% modified hydrotalcites were reduced by 53.64% and 66.19%, separately [12]. Incorporating chitosan (CH)/ammonium polyphosphate (APP) and CH/montmorillonite (MMT) into SiFs, respectively, and found that both CH/APP and CH/MMT could increase the limiting oxygen index (LOI), reduce the peak HRR (PHRR), and smoke production of SiFs [13]. The mechanical properties and flame retardancy of the SiFs composites were significantly improved, and the generation of heat and smoke was inhibited when vinyl-terminated polyborosiloxane filler was mixed into the SiFs matrix [14]. Coating only 0.04 wt% graphene oxide (GO) on the surface of SiFs could significantly reduce its HRR and improve the flame retardancy of SiFs without affecting its density and mechanical flexibility [15]. Sheet and nanoribbon GO coatings were bonded on SiFs and found that GO coatings could improve its thermal stability and flame retardancy without affecting the density and elasticity of SiFs [16]. Furthermore, the fire retardancy and smoke suppression of silicone rubber were also necessary to research [17,18]. The synergies of intumescent flame retardants and Fe_2_O_3_ were very effective methods to improve the flame retardancy and smoke suppression of silicone rubber, Fe_2_O_3_ could improve the smoke suppression efficiency, and thermal degradation temperature of silicone rubber [19]. Furthermore, aluminum hypophosphite and expanded graphite (EG) could effectively improve the flame retardancy and thermal stability of silicone rubber and significantly reduce the production of combustible gas [20].

Sepiolite (SEP) is a kind of natural silicate mineral with the chemical formula of Mg_8_Si_12_O_30_(OH)_4_(H_2_O)_4_·8H_2_O. In its structural unit, the silicon-oxygen tetrahedron and magnesium-oxygen octahedron alternate with each other, with the transitional structure characteristics of layer and chain [21]. Sepiolite (SEP) appeared the demonstrated excellent flame retardancy as a flame retardant from numerous research results [22,23,24,25,26,27]. SEP could significantly enhance the stability of the carbon layer and improve the flame retardancy of silicone rubber [28]. SEP and metal-organic framework were introduced into thermoplastic polyurethane and found the HRR and total heat release (THR) of the composite could be reduced by 78.9% and 39.1%, respectively [29]. SEP/APP/EG flame retardant was introduced into polyethylene octene copolymer, and the experimental results showed that the LOI value could reach 25.3%, and the THR was reduced to 70 MJ/m^2^ [30]. Polypropylene (PP) with 0.5 wt% premodified SEP and 12 wt% commercial intumescent flame retardant could reach UL-94 V-0 classification and effectively improve the flame retardancy and smoke suppression of PP [31]. Waterborne polyurethane (WPU) with SEP and Fe_2_O_3_ had good flame retardancy and thermal stability, and the carbon residue rate was significantly higher than that of pure WPU [32]. Epoxy resin composites with 2.3 wt% a-SEP@layered double hydroxides could reach UL-94 V-1 grade, the LOI value was 31.1%, the peak HRR was reduced by 21%, and the total flue gas production was reduced by 16.0% [33]. However, the direct addition of SEP would cause the mechanical properties degradation of SiFs. The microencapsulation of SEP could effectively improve the compatibility between SEP and SiFs matrix. Thus, the addition of microencapsulated SEP (MSEP) would be an ideal method to improve the flame retardancy and mechanical properties of SiFs.

With the advantages of safety, non-toxicity, and low cost, zinc borate (ZB) has been widely used in the field of polymer flame retardant research and application [34,35,36,37,38]. The synthesis of ZB and SiO_2_ composite could play an influential role in inhibiting fire spread and reducing the THR of silicone rubber [39]. Silicone rubber with aluminum hydroxide, magnesium hydroxide, ZB, and glass frits as additives could reach an LOI value of 34.8%, and the residue of the composite was roughly 58.6% at 700 °C, which was significantly higher than that of pure silicone rubber [40]. ZB and magnesium hydroxide could promote carbon formation and improve the flame retardancy of silicone rubber [41]. Microencapsulated aluminum hypophosphite and ZB could significantly enhance the smoke suppression and flame-retardant properties of SiFs [42]. A total of 3.75% modified diatomite and 1.25% ZB had a positive effect on improving flame retardancy and smoke suppression of SiFs [43]. ZB and a neutralized flame-retardant phosphorus agent exhibited a synergistic effect on the smoke suppression and thermal stability of polypropylene [44]. Moreover, the addition of ZB could significantly improve the thermal stability and self-extinguishing properties of the waste cotton fabric composite aerogel [45].

In this paper, the flame-retardant microcapsules with sepiolite as the core was successfully fabricated. Then, the MSEP was characterized by Fourier Transform Infrared, X-ray photoelectron spectroscopy, scanning electron microscopy, and energy dispersive spectroscopy. Furthermore, the mechanical properties, flame retardance, smoke suppression, pyrolysis characteristics, and carbon residue morphology of SiFs with MSEP/ZB were analyzed.

## 2. Materials and Methods

### 2.1. Materials and Instruments

Materials used in this study were procured as follows: SEP, base gum, and acetic acid were purchased from Xi’an Daosheng Chemical Technology Co., Ltd. (Xi’an, China). Anhydrous ethanol was obtained from Tianjin Kaitong Chemical Reagent Co., Ltd. (Tianjin, China). Ammonia was purchased from Tianjin Damao Chemical Reagent Factory. Tetraethoxysilane (TEOS) was produced by Tianjin Bodi Chemical Co., Ltd. (Tianjin, China). Sodium dodecyl benzene sulfonate (SDBS) was provided by Aladdin Chemical Reagent Co., Ltd. (Shanghai, China). ZB was purchased from Shandong Bio Industry Co, Ltd (Binzhou, China). Hydroxy silicone oil (viscosities: 20–30 MPa s^−1^ and 1500 MPa s^−1^) was produced by Jiangsu Nanoener New Material Co., Ltd. (Nantong, China). Hydroxy silicone oil was purchased from Shandong Dayi Chemical Co., Ltd. (Yantai, China). Pt compounds (3000 ppm) were obtained from Shenzhen Osbang New Materials Co., Ltd. (Shenzhen, China). Methylbutynol was provided by Shaoguan Koya Fine Chemicals Co., Ltd. (Shaoguan, China).

Instruments required for this study were as follows: TD5B desktop large capacity low-speed centrifuge was produced by Changsha Yingtai Instrument Co., Ltd. (Changsha, China). UPH-I-5 high-quality ultrapure water manufacturing system was designed by Xi’an Youpu Instrument Equipment Co., Ltd. (Xi’an, China). STSJB-110 electronic constant speed agitator was purchased from Shanghai Suoyan Electromechanical Equipment Co., Ltd. (Shanghai, China). ESJ 180-4 electronic balance was provided by Shenyang Longteng Electronics Co., Ltd. (Shenyang, China). The 101 vacuum drying oven was obtained by Beijing Kewei Yongxing Instrument Co., Ltd. (Beijing, China). DF-101S collector type constant temperature heating magnetic stirrer was purchased from Gongyi Kerui Instrument Co., Ltd. (Zhengzhou, China).

### 2.2. Preparation of MSEP

**Figure 1:**
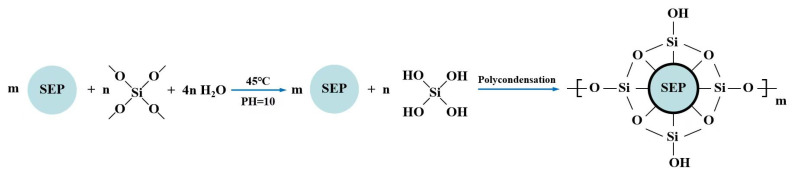
Synthetic process of MSEP.

First, 100 mL deionized water was measured by a 250 mL measuring cylinder and mixed with 1 g SDBS evenly. Then, added the mixture into a 500 mL three-necked flask, heated in an 85 °C water bath, and stirred with a magnetic mixer to dissolve it completely. Subsequently, 3 g SEP was merged, and the solution temperature was kept at 35 °C, stirred at 800 r/min for 2 h, and the SEP emulsion was obtained. Second, 100 mL SEP emulsion, 100 mL anhydrous ethanol, and 10 g TEOS were poured into a three-necked flask and mixed well. Subsequently, an appropriate amount of ammonia was added dropwise to adjust the pH of the solution to 10 at a stirring speed of 100 r/min. The temperature of the water bath was kept at 45 °C for 3 h. Later, cooled the solution to room temperature and adjusted the pH to neutral with 10% acetic acid. Finally, the MSEP was obtained after filtering, washing, and vacuum drying. The synthetic process of MSEP was shown in Figure 1.

### 2.3. Preparation of SiFs with MSEP and ZB

Hydroxy silicone oil, base gum, methylbutynol, Pt compounds, MSEP, and ZB were blended and stirred for 10.0 min. Then, hydrogen-containing silicone oil was added, followed by stirring for 3.0~5.0 min. Subsequently, the mixture was poured into a mold (150.0 × 150.0 × 10.0 mm^3^). Afterward, it foamed and cured at room temperature after about 5.0 min. Finally, the mixture was dried in an oven at 75.0 °C for 5.0 min, and the SiFs were obtained. The formulation of SiFs with MSEP and ZB was shown in Table 1.

### 2.4. Testing and Characterization

The functional group changes of the modified flame retardant were analyzed by Fourier transform infrared spectrometer (Thermo Fisher K-Alpha Plus, Waltham, MA, USA). Before the test, the samples were blended with KBr, and the mixture was compressed into a tablet. The test range was 4000~500 cm^−1^ and the resolution was 4 cm^−1^.

The surface element content and composition of the modified flame retardant were analyzed by X-ray photoelectron spectroscopy (Thermo Fisher K-Alpha Plus, Waltham, MA, USA).

The surface structure of MSEP and the carbon residue structure of SiFs was observed by FEI QUANTA FEG 450 (Hillsboro, OR, USA) scanning electron microscope (SEM). The surface of the sample needed to be sprayed with gold twice in advance with the 20 kV voltage of the test.

The LOI of the SiFs were determined by a JF-3 oxygen index meter (Nanjing Jiangning Analytical Instrument, Co., Ltd., Nanjing, China) in accordance with GB/T 2406-2009, and the sample size was 100 × 5 × 3 mm^3^.

The combustion properties of SiFs were tested using a cone calorimeter (Motis Fire Technology, Co., Ltd., Suzhou, China) in accordance with GB/T16172-2007. The thermal radiation power was 35 kW/m^2^, and the size of the samples was 100 × 100 × 10 mm^3^. All experiments were repeated three times, which result in errors within ±10%.

The smoke density performance test of the SiFs was determined by a JCY-2 smoke density tester (Nanjing Jiangning Analytical Instrument, Co., Ltd., Nanjing, China) in accordance with GB/T 8323v-2008 standard, and the size of the samples was 25 mm × 25 mm × 6 mm.

The tensile and compressive properties of SiFs were tested by HZ-1009C universal tensile testing machine according to ISO 37-2005 standard. The dumbbell-shaped samples were prepared, and the experiments were repeated five times, and the average value was adopted as the elongation at break and tensile strength. The tensile rate was 10 mm/min and the compression rate was 5 mm/min.

Thermogravimetric infrared spectroscopy (TG209F3-TENSOR27, Netzsch-Bruker, Selb, Germany) was used to analyze the thermal decomposition properties and the gas composition after the thermal decomposition of SiFs. At a heating rate of 10 °C/min, the SiFs were heated from 30.0 °C to 900.0 °C in a nitrogen atmosphere. The spectral range and the test range were 4000~500 cm^−1^.

## 3. Results and Discussions

### 3.1. Characterization of MSEP and SEP

**Figure 2:**
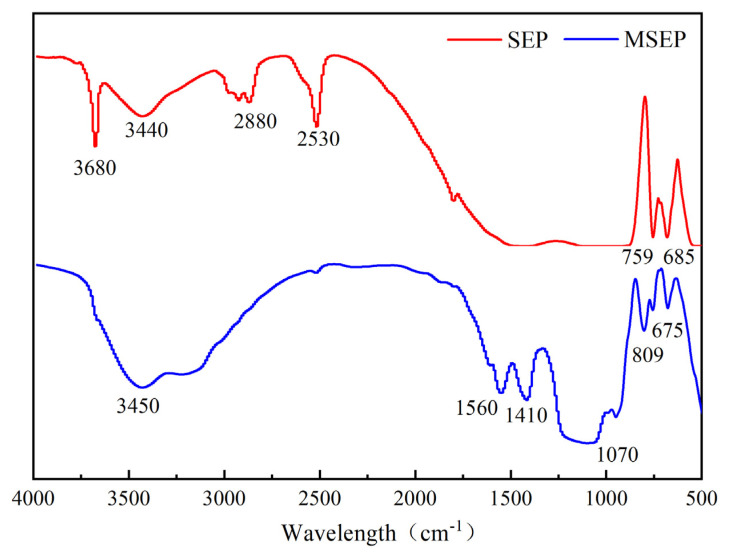
FTIR curves of SEP and MSEP.

The chemical functional group structures of SEP and MSEP were tested by Fourier transform infrared spectra (FTIR). Figure 2 showed that the prominent characteristic absorption peaks of SEP were O-H stretching vibration at 3680 cm^−1^ and 3440 cm^−1^, C-H stretching vibration at 2880 cm^−1^, CO_2_ antisymmetric stretching vibration at 2530 cm^−1^, NH out-of-plane bend at 759 cm^−1^, and out-of-plane bend of the alcohol hydroxyl at 685 cm^−1^. It could be obviously observed that the absorption peak of MSEP around 3450 cm^−1^ was due to the NH_2_ antisymmetric stretching and O-H stretching vibration. Compared with SEP, the absorption peaks at 2880 cm^−1^ and 2530 cm^−1^ disappeared, and three new peaks appeared at 1560 cm^−1^ (in-plane bending of alcohol C-OH), 1410 cm^−1^ (COOH symmetric stretching vibrations), and 1070 cm^−1^ (C-N and C-OH stretching vibration), which indicated that SEP had been successfully coated.

**Figure 3:**
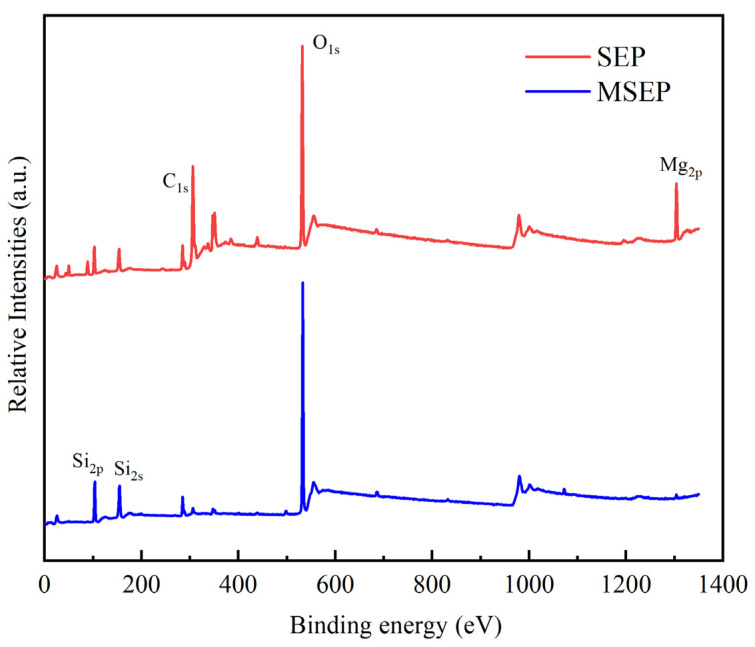
XPS spectra of SEP and MSEP.

The X-ray photoelectron spectroscopy (XPS) spectra and surface elemental compositions of SEP and MSEP were shown in Figure 3 and Table 2. It could be observed that the peak located at 1300 eV was attributed to Mg_2p_ of SEP. The Mg atom content of SEP was 11.79 wt%, while the Mg atom content on the surface of MSEP was little and could not be detected. The Si characterization peak for MSEP was significantly stronger than that of SEP. Furthermore, the Si atom and O atom content of MSEP was 28.23 wt% and 60.64 wt%, respectively, which were 41.79 wt% and 14.61 wt% higher than that of SEP. The above results indicated that SEP was well coated.

**Figure 4:**
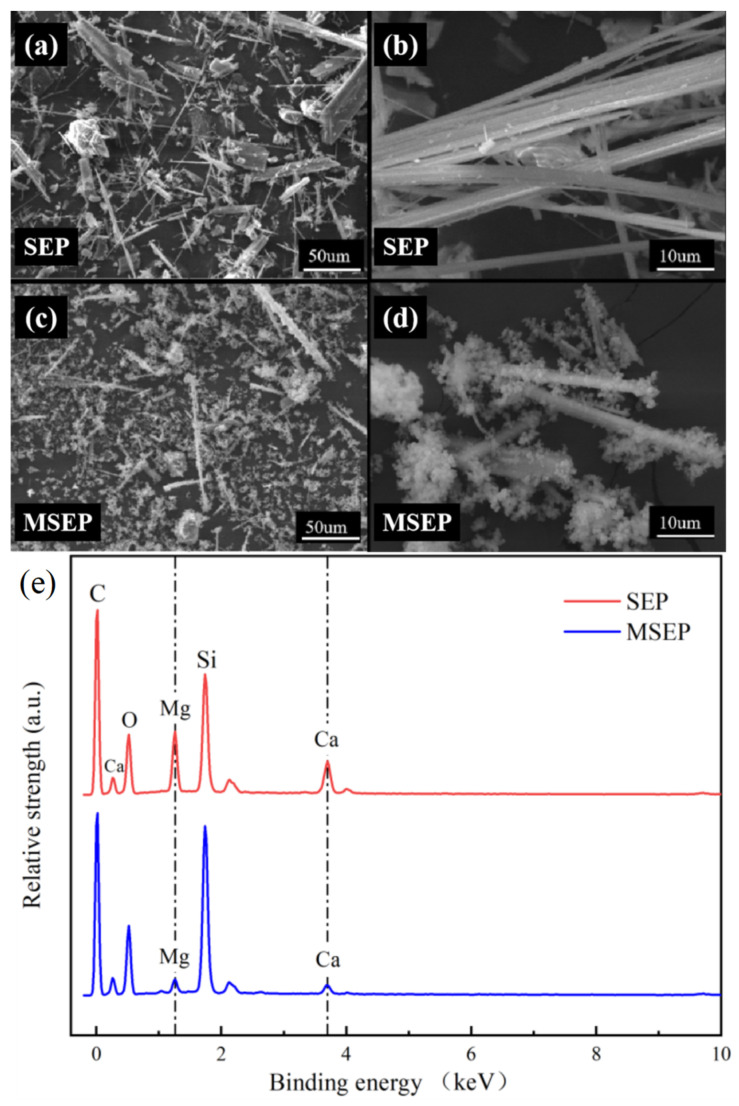
Scanning electron microscopy (**a**–**d**) and energy dispersive spectroscopy images (**e**) of SEP and MSEP.

Scanning electron microscopy and energy dispersive spectroscopy (SEM-EDS) images of MSEP and SEP were shown in Figure 4. Figure 4 showed that SEP was filamentous, the filamentous SEP gathered together, and arranged into sheet structure. However, the surface of MSEP had an obvious flocculent coating. The relative intensities of Ca and Mg elements of MSEP were significantly lower than that of SEP. Moreover, the relative intensities of the Si element were much higher than that of SEP. The SEM-EDS analyses once again proved that SEP was successfully coated.

### 3.2. Mechanical Properties of SiFs with MSEP/ZB

**Figure 5:**
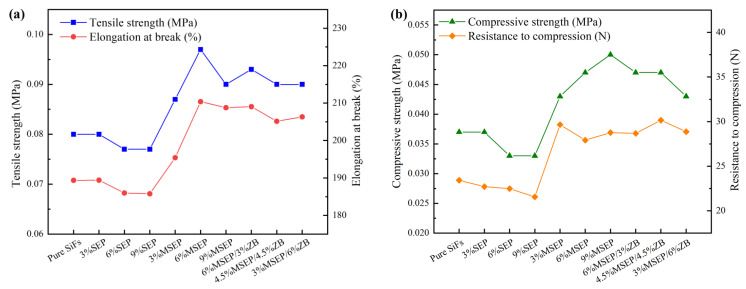
Tensile properties (**a**) and compressive properties (**b**) of SiFs with MSEP and ZB.

The tensile and compressive properties of SiFs with MSEP/ZB were showed in Figure 5. It was clearly found that MSEP and MSEP/ZB could significantly improve the tensile and compressive properties of SiFs. Specifically, the tensile strength, elongation at break, compressive strength, and resistance to compression of SiFs with 9% MSEP were 12.50%, 10.26%, 35.14%, and 22.84% higher than that of pure SiFs. Meanwhile, the tensile strength, elongation at break, compressive strength, and resistance to compression of SiFs with 3% MSEP and 6% ZB were 12.50%, 8.97%, 16.22%, and 23.27% higher than that of pure SiFs. Furthermore, the tensile and compressive properties of SiFs decreased slightly with the increase of SEP, which was due to the poor compatibility between SEP and the matrix material.

### 3.3. Flame Retardant and Smoke Suppression Behaviors of SiFs with MSEP/ZB

The LOI and UL-94 test results of SiFs with MSEP/ZB were shown in Table 3. Notably, the LOI value of SiFs increased with the increase of SEP or MSEP. SiFs with 9 wt% MSEP and SiFs with 3 wt% MSEP and 6 wt% ZB achieved an LOI value of 30.8% and 30.9%, which were 10.8% and 11.2% higher than pure SiFs separately. Furthermore, all the SiFs with flame-retardant fillers could achieve the UL-94-V0 rating.

**Figure 6:**
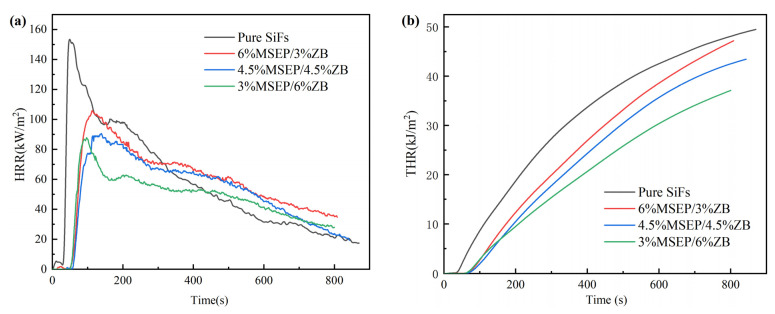
HRR (**a**) and THR (**b**) curves of SiFs with MSEP/ZB.

The related cone calorimeter data of SiFs with MSEP/ZB were listed in Table 4, and the HRR, THR curves of SiFs with MSEP/ZB were shown in Figure 6. The time to ignition (TTI) of SiFs with MSEP/ZB was significantly longer than that of pure SiFs. The TTI of pure SiFs was only 17 s, while the TTI of SiFs with 3 wt% MSEP and 6 wt% ZB increased to 28 s. The PHRR and THR decreased with the increase of ZB when the content of MSEP/ZB was 9 wt%, significantly lower than that of pure SiFs. The PHRR of SiFs with 6 wt% MSEP and 3 wt% ZB, SiFs with 4.5 wt% MSEP and 4.5 wt% ZB, and SiFs with 3 wt% MSEP and 6 wt% ZB were 30.9%, 40.8%, and 42.7% lower than that of pure SiFs. The THR of SiFs with 3 wt% MSEP and 6 wt% ZB was 37.10 MJ/m^2^, which was 25.0% lower than that of pure SiFs. This was possibly due to the compact oxide layer being formed on the surface of SiFs with MSEP/ZB. Furthermore, water vapor was produced and absorbed lots of heat simultaneously when SiFs with MSEP/ZB was heated. The fire performance index (FPI) and fire growth index (FGI) could be used to provide relatively comprehensive assessments of the fire safety of a material [46]. A higher FPI or a lower FGI indicated more excellent fire safety [47,48]. Table 4 exhibited that the FPI obviously raised and the FGI visibly reduced after the incorporation of MSEP/ZB. Specifically, the FPI of pure SiFs was 0.111 m^2^·s/kW, while the FPI of SiFs with 3 wt% MSEP and 6 wt% ZB, SiFs with 4.5 wt% MSEP, and 4.5 wt% ZB could increase to 0.319 m^2^·s/kW and 0.309 m^2^·s/kW, which was 2.87 times and 2.78 times of pure SiFs. Moreover, the FGI of pure SiFs was 3.12 kW/(m^2^·s), while the FGI of SiFs with 3 wt% MSEP and 6 wt% ZB, SiFs with 4.5 wt% MSEP and 4.5 wt% ZB were 28.85%, and 20.83% of pure SiFs.

**Figure 7:**
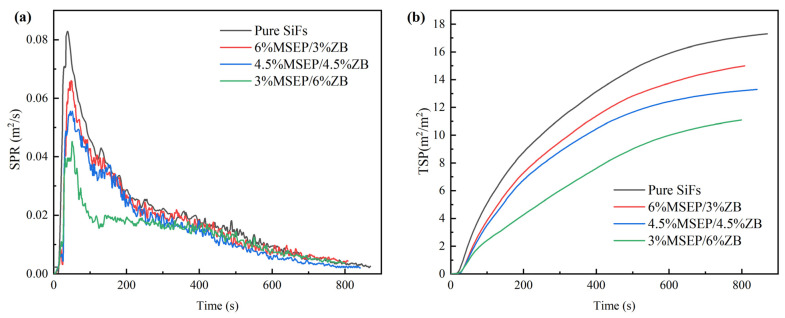
SPR (**a**) and TSP (**b**) curves of SiFs with MSEP/ZB.

The smoke produce rate (SPR) and TSP of SiFs with MSEP/ZB were showed in Figure 7. Figure 7 showed that the SPR and TSP of SiFs with MSEP/ZB were significantly lower than that of pure SiFs. Furthermore, the peak SPR (PSPR) and TSP decreased with the increase of ZB when the content of MSEP/ZB was 9 wt%. Specifically, the PSPR of SiFs with 3 wt% MSEP and 6 wt% ZB was 0.045 m^2^/s, which was 45.78% lower than that of pure SiFs. The TSP of SiFs decreased sharply after the addition of MSEP/ZB. The TSP of SiFs with 3 wt% MSEP and 6 wt% ZB was 11.09 m^2^/m^2^, which was 35.90% lower than that of pure SiFs. This was because MSEP and ZB had synergistic smoke suppression effects, which could promote the formation of solid and dense carbon layers and isolate the transfer of air and heat.

### 3.4. Thermal Decomposition of SiFs with MSEP/ZB

**Figure 8:**
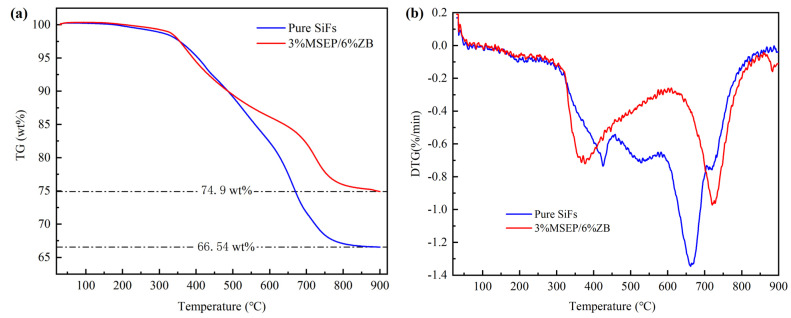
TG (**a**) and DTG (**b**) curves of SiFs with MSEP/ZB in N_2_ atmosphere.

Figure 8 showed the thermogravimetry (TG) and derivative thermogravimetry (DTG) curves of pure SiFs and SiFs with 3 wt% MSEP and 6 wt% ZB in N_2_ atmosphere, and Table 5 showed the thermal degradation parameters of SiFs with MSEP/ZB. It showed that the thermal stability of SiFs was enhanced after the addition of MSEP/ZB. The thermal decomposition process of SiFs composites in the N_2_ atmosphere could be divided into two stages. Furthermore, the maximum decomposition temperature was advanced, and the residual char yield was increased. The residual mass of pure SiFs at 900.0 °C was 66.5%, while the residual mass of SiFs with 3 wt% MSEP and 6 wt% ZB at 900.0 °C could increase to 74.9%. The initial decomposition temperatures (T_-5%_), the first peak mass loss temperature (T_max1_), and the second peak mass loss temperature (T_max2_) of pure SiFs were 404 °C, 423 °C, and 662 °C, respectively. Compared with pure SiFs, the T_max1_ of SiFs with 3 wt% MSEP and 6 wt% ZB was advanced by 55 °C, while the T_max2_ was delayed by 61 °C, and the residual carbon increased by 12.63%, which indicate that the combination of MSEP and ZB could slow the main chain breakage of SiFs. Furthermore, the peak thermal weight loss rates of SiFs with 3 wt% MSEP and 6 wt% ZB were −0.69%/min and −0.94%/min, and the peak temperatures were 368 °C and 723 °C, respectively.

**Figure 9:**
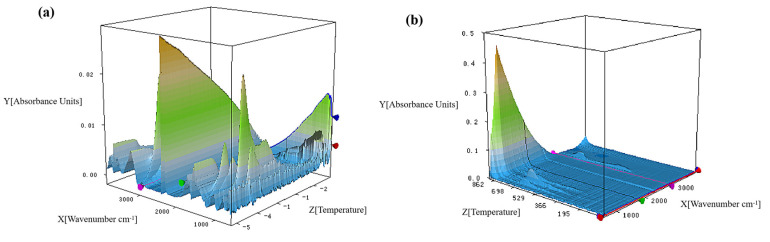
The 3D infrared spectra of pure SiFs (**a**) and SiFs with 3 wt% MSEP and 6 wt% ZB (**b**).

**Figure 10:**
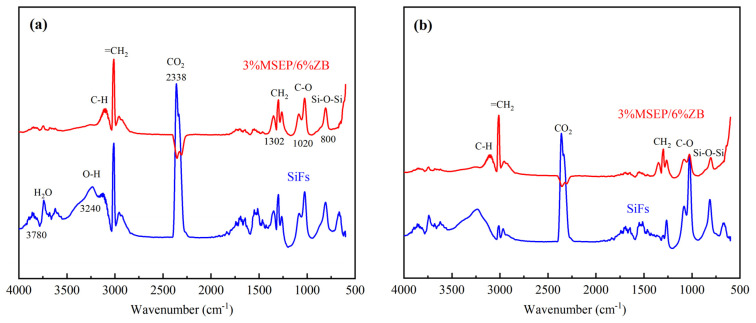
FTIR curves of pure SiFs (**a**) and SiFs with 3 wt% MSEP and 6 wt% ZB (**b**) at T_max1_ and T_max2_.

Figure 9 presented the 3D infrared spectra of pure SiFs and SiFs with 3 wt% MSEP and 6 wt% ZB, and the FTIR spectra of pure SiFs and SiFs with 3 wt% MSEP and 6 wt% ZB at T_max1_ and T_max2_ were presented in Figure 10. The corresponding characteristic absorption peaks of pure SiFs at 3780 cm^−1^, 3240 cm^−1^, and 3000 cm^−1^ were H_2_O vibrational rotation spectra, antisymmetric stretching vibrations of O-H and =CH_2_, and CO_2_ at 2338 cm^−1^, respectively. In addition, the characteristic absorption peaks of SiFs at 1302 cm^−1^ and 1020 cm^−1^ were CH_2_ vibration and C-O stretching. The first maximum thermal weight loss rate of SiFs appeared at around 400 °C, and CO had not yet been released. The contents of CO_2_, CH_4_, and H_2_O of SiFs with 3 wt% MSEP and 6 wt% ZB were all lower than that of pure SiFs. Especially, the CO_2_ content at 2338 cm^−1^ decreased significantly. The C-O absorption peak at T_max2_ was obviously higher than at T_max1_. However, the C-O absorption peak of SiFs with 3 wt% MSEP and 6 wt% ZB was obviously weaker than that of pure SiFs. Notably, the absorption peak of H_2_O of SiFs with 3 wt% MSEP/6 wt% ZB was obviously lower than that of pure SiFs at T_max1_. This may be because the removal of crystal water in MSEP and ZB, and the reinforced carbon layer of SiFs with 3 wt% MSEP and 6 wt% ZB could effectively prevent further oxidation of the side chain of SiFs.

### 3.5. Carbon Residue Morphology Analysis

**Figure 11:**
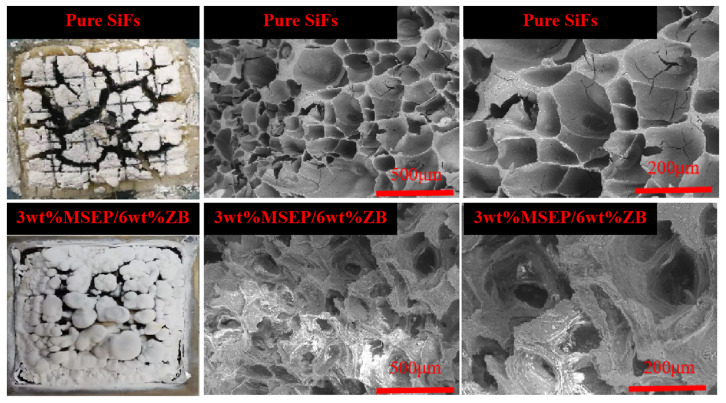
Morphology of carbon residues of SiFs with MSEP/ZB.

The residual carbon morphology of pure SiFs and SiFs with 3 wt% MSEP and 6 wt% ZB were shown in Figure 11. It showed that obvious cracks existed on the surface of the carbon residue of pure SiFs. After adding MSEP/ZB, the carbon residue of SiFs became more compact, and the cracked pores obviously decreased. Notably, SiFs with 3 wt% MSEP and 6 wt% ZB had denser, thicker, and more complete carbon layers, which could act as a protective layer. It could be observed that the white carbon layer on the surface of SiFs with MSEP/ZB increased significantly, and the residual carbon was dense and compact. This was attributed to the fact that the silicon oxide formed by the hydrolysis of tetraethoxysilane was attached to the surface, which could increase the thickness of the carbon layer of the SiFs.

## 4. Conclusions

In this study, MSEP was prepared, and the flame retardancy, smoke suppression behaviors, pyrolysis characteristics, and carbon residue of SiFs with MSEP and ZB were explored. Results demonstrated that MSEP and MSEP/ZB could significantly improve the mechanical properties of SiFs, the LOI value and TTI of SiFs with 6 wt% MSEP and 3 wt% ZB were 11.2% and 64.7% above pure SiFs separately. Moreover, the FPI and FGI of SiFs with 3 wt% MSEP and 6 wt% ZB were 2.87 times and 28.85% of pure SiFs. The PHRR, THR, PSPR, and TSP of SiFs with 3 wt% MSEP and 6 wt% ZB were 42.7%, 25.0%, 45.78%, and 35.90% lower than that of pure SiFs. In addition, the thermal stability of SiFs was evidently improved after the incorporating of 3 wt% MSEP and 6 wt% ZB.

## Figures and Tables

**Table 1:** Formulation of SiFs with MSEP and ZB.

Sample (wt%)	SEP (wt%)	ZB (wt%)	MSEP (wt%)
Pure SiFs	0	0	0
3% SEP	3	0	0
6% SEP	6	0	0
9% SEP	9	0	0
3% MSEP	0	0	3
6% MSEP	0	0	6
9% MSEP	0	0	9
4.5% MSEP/4.5% ZB	0	4.5	4.5
3% MSEP/6% ZB	0	6	3
6% MSEP/3% ZB	0	3	6

**Table 2:** Surface elemental compositions of MSEP and SEP.

Sample	Si (wt%)	O (wt%)	C (wt%)	Mg (wt%)
SEP	19.91	52.91	15.39	11.79
MSEP	28.23	60.64	11.13	/

**Table 3:** Flame retardancy of SiFs with MSEP/ZB or SEP/ZB.

Sample	Density (g cm^−3^)	LOI (%) ± 0.2	UL-94	Dripping
Pure SiFs	0.26	27.8	V1	No
3 wt% SEP	0.26	29.3	V0	No
6 wt% SEP	0.27	29.7	V0	No
9 wt% SEP	0.27	30.2	V0	No
3 wt% MSEP	0.26	29.5	V0	No
6 wt% MSEP	0.27	30.2	V0	No
9 wt% MSEP	0.27	30.8	V0	No
6 wt% MSEP/3 wt% ZB	0.27	30.5	V0	No
4.5 wt% MSEP/4.5 wt% ZB	0.27	30.7	V0	No
3 wt% MSEP/6 wt% ZB	0.27	30.9	V0	No

**Table 4:** Related cone calorimeter data of SiFs with MSEP/ZB.

Sample	TTI (s)	THR (MJ/m^2^)	PSPR (m^2^/s)	TSP (m^2^/m^2^)	PHRR (kW/m^2^)	Time to PHRR (s)	FPI (m^2^·s/kW)	FGI (kW/(m^2^·s))
Pure SiFs	17	49.48	0.083	17.30	153.1	49	0.111	3.12
6 wt% MSEP/3 wt% ZB	25	47.18	0.066	14.99	105.8	114	0.236	0.93
4.5 wt% MSEP/4.5 wt% ZB	28	43.45	0.056	13.30	90.6	139	0.309	0.65
3 wt% MSEP/6 wt% ZB	28	37.10	0.045	11.09	87.7	97	0.319	0.90

**Table 5:** Thermal degradation parameters of SiFs with MSEP/ZB in N_2_ atmosphere.

Sample	T_-5%_ (°C)	T_max1_ (°C)	T_max2_ (°C)	Residual Carbon (%)
Pure SiFs	404	423	662	66.5
3% MSEP/6% ZB	390	368	723	74.9

## Data Availability

The data presented in this study are available on request from the corresponding author. The data are not publicly available due to privacy.

## References

[1] Peng L.G., Lei L., Liu Y.Q., Du L.F. (2021). Improved mechanical and sound absorption properties of open cell silicone rubber foam with NaCl as the pore-forming agent. Materials.

[2] Chruściel J.J., Leśniak E. (2010). Preparation of flexible, self-extinguishing silicone foams. J. Appl. Polym. Sci..

[3] Yang Z., Zhang S.H., Liu W.J., Qi Z.Y. (2021). Preparation and characterization of a composite phase-change material with silicone rubber foam as carrier. Energy Fuel.

[4] Grande J.B., Fawcett A.S., McLaughlin A.J., Gonzaga F., Bender T.P., Brook M.A. (2012). Anhydrous formation of foamed silicone elastomers using the Pierse-Rubinsztajn reaction. Polymer.

[5] Verdejo R., Barroso-Bujans F., Rodriguez-Perez M.A., Saja J.A., Lopez-Manchado M.A. (2008). Functionalized graphene sheet filled silicone foam nanocomposites. J. Mater. Chem..

[6] Liu X.X., Ma L., Sheng Y.J., Liu S.M. (2022). Synergistic flame-retardant effect of modified hydrotalcite and expandable graphite for silicone rubber foam. J. Appl. Polym. Sci..

[7] Liu P., Liu D.L., Zou H.W., Fan P., Xu W. (2009). Structure and properties of closed-cell foam prepared from irradiation crosslinked silicone rubber. J. Appl. Polym. Sci..

[8] Song J.Q., Huang Z.X., Qin Y., Yang G.Y., Wang X. (2018). Ceramifiable and mechanical properties of silicone rubber foam composite with frit and high silica glass fiber. IOP Conf. Ser. Mater. Sci. Eng..

[9] Han Y.G., Yang L.J., Yu Z., Zhao Y.J., Zhang Z.X. (2023). Lightweight and flame retardant silicone rubber foam prepared by supercritical nitrogen: The influence of flame retardants combined with ceramicizable fillers. Constr. Build. Mate..

[10] Song L.X., Lu A., Feng P.J., Lu Z.Y. (2014). Preparation of silicone rubber foam using supercritical carbon dioxide. Mater. Lett..

[11] Zhu C., Deng C., Cao J.Y., Wang Y.Z. (2015). An efficient flame retardant for silicone rubber: Preparation and application. Polym. Degrad. Stabil..

[12] Ma L., Liu X.X., Sheng Y.J., Guo Y. (2022). Flame retardancy of silicone rubber foam containing modified hydrotalcite. J. Appl. Polym. Sci..

[13] Deng S.B., Liao W., Yang J.C., Cao Z.J., Wang Y.Z. (2016). Flame-retardant and smoke-suppressed silicone foams with chitosan-based nanocoatings. Ind. Eng. Chem. Res..

[14] Du W.N., Yin C.L., Huang H., Ge X.G. (2022). Vinyl-functionalized polyborosiloxane for improving mechanical and flame-retardancy performances of silicone rubber foam composites. Polym. Int..

[15] Guo B.F., Wang P.H., Cao C.F., Qu Z.H., Lv L.Y., Zhang G.D., Gong L.X., Song P., Gao J.F., Mai Y.W. (2022). Restricted assembly of ultralow loading of graphene oxide for lightweight, mechanically flexible and flame retardant polydimethylsiloxane foam Composites. Compos. Part B Eng..

[16] Cao C.F., Wang P.H., Zhang J.W., Guo K.Y., Li Y., Xia Q.Q., Zhang G.D., Zhao L., Chen H., Wang L. (2020). One-step and green synthesis of lightweight, mechanically flexible and flame-retardant polydimethylsiloxane foam nanocomposites via surface-assembling ultralow content of graphene derivative. Chem. Eng. J..

[17] Hermansson A., Hjertberg T., Sultan B. (2010). Distribution of calcium carbonate and silicone elastomer in a flame retardant system based on ethylene-acrylate copolymer, chalk and silicone elastomer and its effect on flame retardant properties. J. Appl. Polym. Sci..

[18] Hamdani-Devarennes S., Longuet C., Sonnier R., Ganachaud F., Lopez-Cuesta J.-M. (2013). Calcium and aluminum-based fillers as flame-retardant additives in silicone matrices. III. investigations on fire reaction. Polym. Degrad. Stab..

[19] Chen X.L., Li M., Zhuo J.L., Ma C.Y., Jiao C.M. (2015). Influence of Fe_2_O_3_ on smoke suppression and thermal degradation properties in intumescent flame-retardant silicone rubber. J. Therm. Anal. Calorim..

[20] Chen X.L., Song W.K., Liu J.B., Jiao C.M., Qian Y. (2015). Synergistic flame-Retardant effects between aluminum hypophosphite and expandable graphite in silicone rubber composites. J. Therm. Anal. Calorim..

[21] Chen B., Jia Y., Zhang M., Li X., Yang J., Zhang X. (2019). Facile modification of sepiolite and its application in superhydrophobic coatings. Appl. Clay Sci..

[22] Li X., Liang D., Hu Z.Y., He J.L., Bian X.C., Cui J.L. (2021). Synergistic effects of polyoxometalate–based ionic liquid–doped sepiolite in intumescent flame–retardant high–density polyethylene. Polym. Adv. Technol..

[23] Li W.X., Li S.X., Cheng Z., Hu X.P., Yang W.B., Yao Y. (2019). The effect of flame retardant-modified sepiolite nanofibers on thermal degradation and fire retardancy of low-density polyethylene. J. Therm. Anal. Calorim..

[24] Zhang C.Y., Wang J.C., Song S.Q. (2019). Preparation of a novel type of flame retardant diatomite and its application in silicone rubber composites. Adv. Powder Technol..

[25] Xu Z.S., Liu D.L., Yan L., Xie X.J. (2020). Synergistic effect of sepiolite and polyphosphate ester on the fire protection and smoke suppression properties of an amino transparent fire-retardant coating. Prog. Org. Coat..

[26] Huang N.H., Chen Z.J., Yi C.H., Wang J.Q. (2010). Synergistic flame retardant effects between sepiolite and magnesium hydroxide in ethylene-vinyl acetate (EVA) matrix. Express Polym. Lett..

[27] Nguyen Thanh T.T., Szolnoki B., Vadas D., Nacsa M., Marosi G., Bocz K. (2022). Effect of clay minerals on the flame retardancy of polylactic acid/ammonium polyphosphate system. J. Therm. Anal. Calorim..

[28] Liu J.Q., Xin Z.X. (2023). Effect of sepiolite on properties of silicone rubber/melamine/starch/sepiolite flame retardant composites. J. Appl. Polym. Sci..

[29] Li H.F., Meng D., Qi P., Sun J., Li H.F., Gu X.Y., Zhang S. (2022). Fabrication of a hybrid from metal organic framework and sepiolite (ZIF-8@SEP) for reducing the fire hazards in thermoplastic polyurethane. Appl. Clay Sci..

[30] Zhang Y.F., Yu C.M., Feng X. (2022). Simultaneously improving flame retardant performance, thermal stability and conductivity of copolymers of polyethylene-octene by addition of a ternary composite flame retardant system. ChemistrySelect.

[31] Pappalardo S., Russo P., Acierno D., Rabe S., Schartel B. (2016). The synergistic effect of organically modified sepiolite in intumescent flame retardant polypropylene. Eur. Polym. J..

[32] Xu T., Qian D., Hu Y.L., Zhu Y.Z., Zhong Y., Zhang L.P., Xu H., Peng H.X., Mao Z.P. (2021). Effect of sepiolite-loaded Fe_2_O_3_ on flame retardancy of waterborne polyurethane. Adv. Polym. Technol..

[33] Zhang H.J., Hu X.P., Liu Y.R., Zhang S.H., Wu Z.Z. (2021). Convenient synthesis of one-dimensional a-SEP@LDH via self-Assembly towards simultaneously improved fire retardance, mechanical strength and thermal resistance for epoxy resin. Compos. Part B Eng..

[34] Sultygova Z.K., Kitieva L.I., Timur A., Borukaev T.A. (2019). Using zinc borate as effective flame retardant. Key Eng. Mater..

[35] Jiang M.W., Zhang Y.S., Yu Y., Zhang Q.W., Huang B.J., Chen Z.Q., Chen T.T., Jiang J.C. (2019). Flame retardancy of unsaturated polyester composites with modified ammonium polyphosphate, montmorillonite, and zinc borate. J. Appl. Polym. Sci..

[36] İPek Y. (2020). Effect of surfactant types on particle size and morphology of flame-retardant zinc borate powder. Turk. J. Chem..

[37] Sertsova A.A., Marakulin S.I., Yurtov E.V. (2017). Metal compound nanoparticles: Flame retardants for polymer composites. Russ. J. Gen. Chem..

[38] Mahajan D.S., Deshpande T.D., Bari M.L., Patil U.D., Narkhede J.S. (2020). Hydrated and anhydrous zinc borate fillers for tuning the flame retardancy of epoxy nanocomposites. J. Appl. Polym. Sci..

[39] Wang X.S., Li L., Tong Y.J., Dai Y., Chen W.D. (2020). Synthesis of core/shell structured zinc borate/silica and its surface charring for enhanced flame retardant properties. Polym. Degrad. Stabil..

[40] Li Z.X., Liang W.J., Shan Y.F., Wang X.X., Yang K., Cui Y.Y. (2020). Study of flame-retarded silicone rubber with ceramifiable property. Fire Mater..

[41] Cheng X., Wu J.M., Yao C.G., Yang G.S. (2019). Flame-retardant mechanism of zinc borate and magnesium hydroxide in aluminum hypophosphite-based combination for TPE-S composites. J. Fire Sci..

[42] Kang F.R., Wang C.P., Deng J., Yang K., Pang Q.T. (2020). Flame retardancy and smoke suppression of silicone foams with microcapsulated aluminum hypophosphite and zinc borate. Polym. Adv. Technol..

[43] Wang C.P., Qiao X.T., Kang F.R., Li H., Deng J., Xiao Y., Lv C.H. (2022). Flame Retardancy and Smoke Suppression of Silicone Foams with Modified Diatomite and Zinc Borate. Combust. Sci. Technol..

[44] Fontaine G., Bourbigot S., Duquesne S. (2008). Neutralized flame retardant phosphorus agent: Facile synthesis, reaction to fire in PP and synergy with zinc borate. Polym. Degrad. Stabil..

[45] Qin Q., Guo R.H., Ren E.H., Lai X.X., Cui C., Xiao H.Y., Zhou M., Yao G., Jiang S.X., Lan J.W. (2020). Waste cotton fabric/zinc borate composite aerogel with excellent flame retardancy. ACS Sustain. Chem. Eng..

[46] Nazare S., Kandola B., Horrocks R. (2002). Use of cone calorimetry to quantify the burning hazard of apparel fabrics. Fire Mater..

[47] Jiao C.M., Zhao X.L., Song W.K., Chen X.L. (2015). Synergistic flame retardant and smoke suppression effects of ferrous powder with ammonium polyphosphate in thermoplastic polyurethane composites. J. Therm. Analy. Calorim..

[48] Schartel B., Hull T.R. (2010). Development of fire retarded materials-interpretation of cone calorimeter data. Fire Mater..

